# Nutritional status in patients with chronic pancreatitis and liver cirrhosis is related to disease conditions and not dietary habits

**DOI:** 10.1038/s41598-024-54998-7

**Published:** 2024-02-26

**Authors:** Niklas Bruns, Fatuma Meyer, Karen Rischmüller, Fabian Frost, Quang Trung Tran, Till Ittermann, Martin Bahls, Luzia Valentini, Georg Lamprecht, Markus M. Lerch, Ali A. Aghdassi, Mats L. Wiese

**Affiliations:** 1 Department of Medicine A, University Medicine Greifswald, Ferdinand-Sauerbruch-Straße, 17475 Greifswald, Germany; 2 Institute of Evidence-Based Dietetics (NIED), University of Applied Sciences Neubrandenburg, Neubrandenburg, Germany; 3 Division of Gastroenterology, Department of Medicine II, Rostock University Medical Center, Rostock, Germany; 4 Institute for Community Medicine, University of Greifswald, Greifswald, Germany; 5 Department of Internal Medicine B, University Medicine Greifswald, Greifswald, Germany; 6 LMU University Hospital, Ludwig Maximilian Universität München, Munich, Germany

**Keywords:** Chronic pancreatitis, Liver cirrhosis, Malnutrition, Diet, Habitual food intake, Gastroenterology, Nutrition

## Abstract

Malnutrition is a common complication of chronic pancreatitis (CP) and liver cirrhosis (LC). Inadequate food intake is considered a relevant driver of malnutrition in both entities. However, the contribution of habitual diet to impaired nutritional status is unclear. In a prospective, multicenter cross-sectional study, we recruited patients with confirmed CP or LC and healthy volunteers as a control group. Malnutrition was diagnosed according to the Global Leadership Initiative on Malnutrition criteria. We comprehensively investigated habitual dietary intake on nutrient, food group, and dietary pattern level applying two validated food frequency questionnaires. We included 144 patients (CP: n = 66; LC: n = 78) and 94 control subjects. Malnutrition was prevalent in 64% and 62% of patients with CP or LC, respectively. In both CP and LC, despite slightly altered food group consumption in malnourished and non-malnourished patients there were no differences in energy or nutrient intake as well as dietary quality. Compared to controls patients showed distinct dietary food group habits. Patients consumed less alcohol but also lower quantities of fruits and vegetables as well as whole grain products (*p* < 0.001, respectively). Nevertheless, overall dietary quality was comparable between patients and healthy controls. Nutritional status in CP and LC patients is rather related to disease than habitual dietary intake supporting the relevance of other etiologic factors for malnutrition such as malassimilation or chronic inflammation. Despite distinct disease-related differences, overall dietary quality in patients with CP or LC was comparable to healthy subjects, which suggests susceptibility to dietary counselling and the benefits of nutrition therapy in these entities.

## Introduction

Malnutrition is a common sequel of chronic gastrointestinal diseases leading to reduced quality of life, more frequent hospitalizations, greater risk of complications, and if untreated, increased morbidity and mortality^1–5^. Chronic pancreatitis (CP) and liver cirrhosis (LC) are two chronic gastrointestinal diseases, which are particularly associated with an increased malnutrition risk^6–8^. Alcohol abuse is an important etiologic factor for both entities that may cause decreased food intake and enhanced nutrient losses^9–13^.

In CP, pancreatic function is gradually being lost due to the fibrotic replacement of functional pancreatic tissue resulting from recurrent inflammatory episodes^14^. The global prevalence of CP is estimated at around 50 per 100,000 persons^15^. CP can cause severe abdominal pain, exocrine pancreatic insufficiency and pancreatogenic diabetes, which may impair food intake and digestion resulting in weight loss, nutrient deficiencies and ultimately malnutrition^8,16,17^.

LC is the late-stage of liver disease with a prevalence between 500 and 1100 per 100,000 inhabitants in European countries^18^. Malnutrition in LC may result from poor dietary intake, fat malabsorption due to reduced bile acid synthesis, increased intestinal protein losses, decreased hepatic protein synthesis and glycogen storage capacity, poor substrate utilization and hypermetabolism^7,19^. Malnourished patients with LC are at greater risk of infections, portal hypertension and its associated complications^19,20^.

The Global Leadership Initiative on Malnutrition (GLIM) international consensus criteria recognize reduced food intake as a well-established and valid etiologic criterion for the diagnosis of malnutrition^21^. However, other etiologic factors, namely impaired food or nutrient assimilation, as well as acute or chronic inflammation, can further deteriorate nutritional status. As of today, it is unknown to what extent these factors contribute to the development of malnutrition in specific diseases. Our previous works suggest that malnutrition in CP and LC may occur despite unchanged food intake, which has relevant implications for the treatment of malnourished patients with these diseases^22,23^. Therefore, in this prospective study we comprehensively investigated the association between nutritional status of patients with CP or LC and their habitual dietary intake on a nutrient, food group, and dietary pattern level.

## Materials and methods

### Study design and setting

Data for this multicenter cross-sectional study was prospectively collected from October 2018 to September 2021 in the federal state of Mecklenburg-Vorpommern, located in the Northeast of Germany with a population of around 1.6 million inhabitants. Patients with CP or LC were enrolled at University Medicine Greifswald and Rostock University Medical Center, two tertiary referral centers in that area. As a control group, healthy volunteers were recruited from the general population at University of Applied Sciences Neubrandenburg.

The study was approved by the respective local Institutional Review Boards (internal registration numbers: A 2018-0129, BB 155/18, HSNB/AL/143/18) and registered at clinicaltrials.gov (NCT04474743). All study-related procedures were carried out in accordance with the ethical principles related to the Declaration of Helsinki.

### Subjects

All subjects were over 18 years of age and neither pregnant nor lactating to be eligible for study participation. Diagnosis of CP was confirmed by imaging modality, based on either endoscopic ultrasound, computed tomography or magnetic resonance imaging combined with magnetic resonance cholangiopancreatography and histology if available. Likewise, LC was diagnosed based on indicative imaging or histology findings. Patients were further ineligible, if they had been diagnosed with any malignant disease within the last 3 years or any other severe chronic gastrointestinal disease. Subjects with concomitant diagnosis of CP and LC were excluded from the analyses.

Disease severity was graded based on the Chronic Pancreatitis Prognosis Score (COPPS)^24^ for CP and for LC we used the clinically established Child–Pugh Score^25^.

Healthy volunteers were required to be free of any acute or chronic disease, be at a good health status, equivalent to an Eastern Cooperative Oncology Group Status of 0, have a body mass index (BMI) between 18.5 and 34.9 kg/m^2^ as well as weight stability for at least 6 months.

### Diagnosis of malnutrition

Malnutrition was diagnosed in accordance with international consensus criteria, proposed by the Global Leadership on Malnutrition (GLIM)^21^. These criteria require the presence of at least one phenotypic and one etiologic criterion. Phenotypic criteria compromise non-volitional weight loss, low BMI and reduced muscle mass. On the other hand, reduced food intake or assimilation and inflammation related to chronic disease, acute disease or injury are considered as etiologic criteria. We performed bioelectrical impedance analysis (BIA, seca mBCA 515 and mBCA 525, seca, Hamburg, Germany) for measurement of muscle mass in all subjects. Actual body weight including ascites or edema was used to calculate the BMI by dividing body mass by height squared (kg/m^2^). To evaluate fulfillment of any of the phenotypic or etiologic criteria, we used thresholds as recommended by the GLIM.

### Dietary assessment

We used two different food frequency questionnaires (FFQs) to collect comprehensive data on habitual food consumption. A validated short-form qualitative FFQ (SHIP-FFQ) inquired the frequencies of consumption for various food groups^26^. Based on the consumption of 15 selected food groups a composite score reflecting dietary quality was derived, as previously described in detail^27^. Briefly, for this SHIP-FFQ score a value of 0, 1 or 2 points was assigned to each food group. Subsequently, the sum over all groups was calculated to derive the final score, with a maximum value of 30 points indicating perfect agreement with the recommendations of the German Society of Nutrition^28^. As a second instrument, we employed a validated semi-quantitative FFQ (DEGS-FFQ) to obtain information on subjects’ intake of energy and nutrients^29^. The DEGS-FFQ asked study participants about the consumption of 53 food items in the preceding four weeks. Daily consumption of each food item was derived by multiplying the monthly frequency of consumption by the portion size and dividing by 28. Daily intake of energy, macro- and micronutrients was then calculated by multiplying the quantity of daily consumption with average nutrient composition of each food item. Energy and protein intake were reported both as absolute numbers and in relation to body weight. To account for the presence of ascites, edema, or obesity, we calculated relative intakes based on ideal body weight, referred to a BMI of 22 kg/m^2^, in these subjects^30,31^. Information on nutrient composition was obtained from the German Nutrient Database *(“Bundeslebensmittelschlüssel”)*^32^. Besides energy and nutrient intake, we calculated the DEGS-Healthy Eating Index (DEGS-HEI), which evaluates dietary quality by also considering quantity of consumption, as previously described^33^. The DEGS-HEI ranges from 0 to 100 and a higher score reflects a more favorable quality of diet.

### Statistical analysis

Continuous data were presented as mean (± standard deviation, SD) or median and (interquartile range, IQR) based on normality of their distribution; categorical data were shown as absolute number (n) and relative percentage (%). Normality of distribution for continuous variables was assessed using the Kolmogorov–Smirnov (n ≥ 50) or Shapiro–Wilk test (n < 50) in combination with visual inspection of the Q-Q and residual plots. With regard to inferential statistics, we used two-sided t-test to compare normally distributed continuous variables between groups and the Mann–Whitney U test or Kruskal–Wallis test, in case of non-normal distribution of data. When we found significant differences, we applied Bonferroni corrected post-hoc testing for pairwise comparison between individual groups. Chi-squared test was applied to compare categorical variables. When testing multiple hypotheses, we applied Bonferroni correction to account for multiple testing. A *p*-value < 0.05 defined statistical significance. All statistical analyses were performed using IBM SPSS Statistics for Windows version 28 (IBM Corp., Armonk, NY, United States).

### Ethical approval

This study was reviewed and approved by the Local Ethics Committees of University Medicine Greifswald (BB 155/18), Rostock University Medical Center (A 2018–0129), and University of Applied Sciences Neubrandenburg (HSNB/AL/143/18). All study-related procedures were conducted in accordance with the ethical principles of the Declaration of Helsinki.

### Informed consent

All participants provided their informed written consent.

## Results

### Subject enrollment and participant characteristics

**Figure 1:**
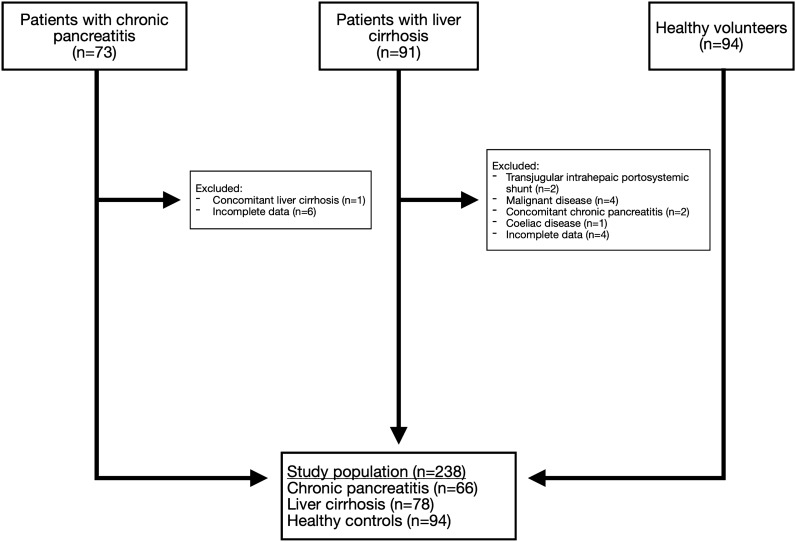
Flowchart illustrating the selection of the study population.

Initially, 73 patients with CP, 91 patients with LC and 94 healthy volunteers were evaluated in the study. Of these, 20 patients (CP: n = 7; LC: n = 13) were excluded for the final analyses because of a concomitant malignant disorder, co-existing CP or LC, diagnosis of a bowel disease (coeliac disease), placement of a transjugular intrahepatic portosystemic shunt or missing values **(**Fig. 1**)**. Thus, the final study population consisted of 238 subjects, including 66 patients with CP, 78 patients with LC and 94 healthy volunteers.

Basic demographic and clinical participant characteristics are presented in Table 1. While age was comparable between groups, there was a significant difference in sex distribution (*p* < 0.001) with higher proportion of males among patients with CP and LC compared to controls, respectively.

**Table 1:** Basic demographic and clinical participant characteristics.

	Chronic pancreatitis (n = 66)	Liver cirrhosis (n = 78)	Healthy controls (n = 94)	
Age, years^a^	59 (19)	57 (11)	54 (16)	
Sex, n (%)				
Female	15 (23)	26 (33)	49 (52)	#
Male	51 (77)	52 (67)	45 (48)	#
Etiology, n (%)				
Alcohol	28 (42)	58 (74)	–	
Autoimmune	2 (3)	7 (9)	–	
Idiopathic/cryptogenic	36 (55)	2 (3)	–	
Steatohepatitis/NASH	–	4 (5)	–	
Primary biliary cirrhosis	–	2 (3)		
Chronic hepatitis C	–	1 (1)	–	
Other genesis^b^	–	4 (5)	–	
Disease severity, n (%)				
COPPS / CHILD A	20 (30)	15 (19)	–	
COPPS / CHILD B	35 (53)	32 (41)	–	
COPPS / CHILD C	11 (17)	31 (40)	–	
Ascites	2 (3)	61 (78)	–	
Edema	3 (5)	37 (47)	–	
Continued substance use, n (%)				
Alcohol	21 (32)	24 (31)	86 (91)	#
Nicotine	31 (47)	26 (33)	12 (13)	#
Nutritional status				
Malnutrition according to GLIM, n (%)	42 (64)	48 (62)	–	
BMI^a^, kg/m^2^	25 (7)	27 (7)	26 (4)	§
BMI categories, n (%)				
BMI < 20 kg/m^2^	8 (12)	4 (5)	5 (5)	
20 kg/m^2^ ≤ BMI < 25 kg/m^2^	25 (38)	23 (30)	36 (38)	
25 kg/m^2^ ≤ BMI < 30 kg/m^2^	17 (26)	28 (36)	39 (42)	
BMI ≥ 30 kg/m^2^	16 (24)	23 (30)	14 (15)	
Weight loss (past 3 months), n (%)	20 (30)	37 (47)	–	‡
NRS-2002 ≥ 3, n (%)	19 (29)	38 (49)	–	‡

COPPS, Chronic Pancreatitis Prognosis Score; NRS-2002, Nutritional Risk Screening 2002*.*

^a^Data is presented as median (IQR).

^b^Trauma/shock-related genesis, Budd-Chiari syndrome, alpha-1-antitrypsin deficiency, primary sclerosing cholangitis.

Differences between continuous variables were tested using Kruskal–Wallis test.

Differences between categorical variables were tested using χ2-test.

^#^Significant difference between patients with chronic pancreatitis or liver cirrhosis and healthy controls (*p* < 0.001).

^§^Significant difference between patients with liver cirrhosis and healthy controls (*p* < 0.05).

^‡^Significant difference between patients with chronic pancreatitis and patients with liver cirrhosis (*p* < 0.05).

Alcohol abuse was the most common etiology of LC (74%) and the second most in patients with CP (42%), in which idiopathic CP (55%) was slightly more prevalent. While approximately one third of patients (CP: 32%; LC: 31%) reported continued alcohol consumption, the proportion of alcohol drinkers (91%) was significantly higher among healthy controls (*p* < 0.001). Conversely, smoking was more common in patients with CP (47%) or LC (33%) as compared to controls (13%, *p* < 0.001). With regard to nutritional status, malnutrition was diagnosed in 64% of patients with CP and in 62% of patients with LC. While BMI was comparable between CP patients and controls, median BMI in patients with LC was significantly higher than among healthy volunteers (*p* = 0.044). Yet, in comparison to subjects with CP, patients with LC more often reported weight loss in the past 3 months (*p* = 0.043) and at a higher rate screened positive for malnutrition risk identified by Nutritional Risk Screening 2002 (NRS-2002) (*p* = 0.018).

### Relation between habitual dietary intake and nutritional status

**Figure 2:**
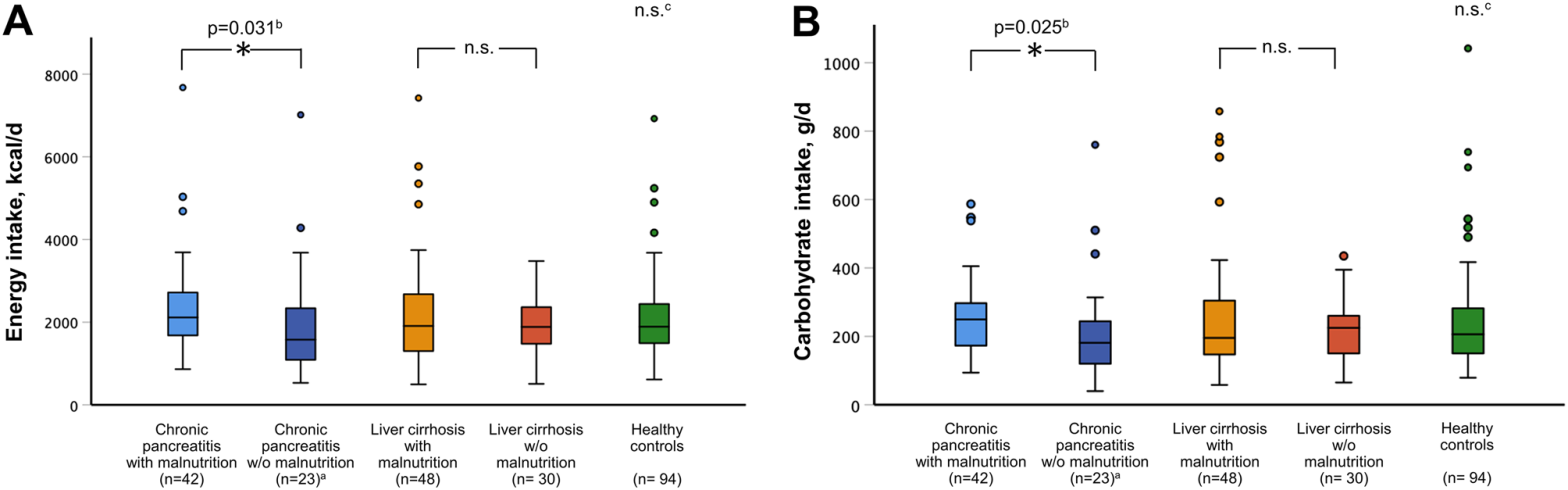
Comparison of daily energy and carbohydrate intakes. Daily intakes of energy (**A**) and carbohydrates (**B**) in patients with chronic pancreatitis or liver cirrhosis with or without malnutrition and healthy controls. The p-values for comparison were obtained by Mann–Whitney U test. ^a^one patient did not complete the food frequency questionnaire and was excluded from analysis; ^b^*p*-values > 0.05 after stratification by sex; n.s., not significant; n.s.^c^=no significant differences for comparison with any other group.

When we compared dietary habits of malnourished and non-malnourished patients with CP or LC, we found no substantial differences for energy or nutrient intake. In unstratified analyses, patients with CP and concurrent diagnosis of malnutrition showed significantly higher energy intake than non-malnourished patients [2114 kcal/d vs. 1576 kcal/d, *p* = 0.031] while no difference existed in LC patients (Fig. 2A). Similar results were obtained for carbohydrate consumption (*p* = 0.025), the main source for energy supply among patients (Fig. 2B). However, after stratification by sex this difference was no longer significant. Regarding the intake of macro- and micronutrients we could not find any further significant differences (Supplementary Table 1). Comparison of food group consumption yielded few significant differences for some food items between malnourished and non-malnourished patients (Supplementary Table 2). These included an increased intake of lemonade (*p* = 0.027), tea (*p* = 0.013), white bread (*p* = 0.006), boiled potatoes (*p* = 0.041) and desserts and sweet spreads (*p* = 0.017) in malnourished patients with CP compared to non-malnourished individuals whereas malnourished LC patients consumed lower quantities of eggs and meat and poultry than non-malnourished patients (*p* = 0.008 and *p* = 0.017). After stratification by sex, most differences, however, remained significant only in male patients. These comprised higher intakes of tea and desserts and sweet spreads in malnourished CP patients as well as reduced intake of eggs in malnourished LC patients (Supplementary Tables 3 and 4).

Likewise, the dietary quality indices for both CP and LC were similar between malnourished and non-malnourished patients (Supplementary Table 5). Only dietary quality assessed by SHIP-FFQ was significantly lower in malnourished than in non-malnourished patients with LC [11.91 (± 3.12) vs 13.43 (± 3.45), *p* = 0.049]. Yet again, this difference no longer subsisted after stratification by sex.

### Relation between habitual dietary intake and disease

Unlike nutritional status, we found deviations in habitual dietary intake between patients with CP or LC and healthy controls. These differences were most notably seen on the micronutrient level, but there were also some distinctions with regard to macronutrient intake.

Table 2 shows the daily intakes of energy and macronutrients for patients with CP or LC and for healthy controls. The consumption of protein, carbohydrates, fat and fatty acids was comparable between groups. By contrast, there were significant differences in alcohol intake. Both patient groups consumed less alcohol compared to healthy controls (*p* < 0.001) while intake did not differ between the two entities. In addition, patients with LC also drank lower amounts of water (*p* = 0.019) and consumed less dietary fiber (*p* < 0.001) than healthy controls whereas intake was comparable to patients with CP. When performing sex-stratified analyses, we confirmed these differences in alcohol and dietary fiber consumption in both male and female patients. Additionally, only male subjects with LC had a lower consumption of polyunsaturated fatty acids compared to healthy controls (*p* = 0.043) (Supplementary Table 6).

**Table 2:** Energy and macronutrient intake in patients with chronic pancreatitis or liver cirrhosis and healthy controls.

	Chronic pancreatitis (n = 65)^a^	Liver cirrhosis (n = 78)	Healthy controls (n = 94)	*p*-value^b^	*p*-value^c^	*p*-value^d^
Energy, kcal/d	1915 (1121)	1893 (1161)	1889 (953)	1.000	1.000	1.000
Energy, kcal/kg body weight^e^/d	25.4 (19.6)	28.0 (16.7)	26.2 (14.1)	1.000	0.916	1.000
Protein, g/d	76 (39)	66 (50)	76 (40)	1.000	0.249	0.425
Protein g/kg body weight^e^/d	1.1 (0.7)	1.0 (0.7)	1.0 (0.5)	1.000	1.000	1.000
Fat, g/d	76 (55)	58 (54)	63 (36)	0.740	1.000	0.413
Saturated fatty acids, g/d	38 (30)	28 (25)	32 (21)	0.665	1.000	0.476
Monounsaturated fatty acids, g/d	27 (21)	20 (19)	23 (14)	0.607	1.000	0.297
Polyunsaturated fatty acids, g/d	10 (8)	8 (9)	10 (6)	1.000	0.061	0.342
Cholesterol, g/d	289 (222)	256 (266)	291 (146)	1.000	1.000	0.435
Carbohydrates, g/d	221 (130)	201 (124)	206 (133)	1.000	1.000	1.000
Alcohol, g/d	0 (4)	0 (8)	6 (12)	** < 0.001**	** < 0.001**	1.000
Dietary fiber, g/d	21 (15)	19 (14)	25 (17)	0.079	** < 0.001**	0.608
Water, ml/d	2765 (1807)	2692 (1428)	2990 (4075)	0.816	**0.019**	0.448

All data is presented as median (IQR); bold typed numbers indicate *p*-value < 0.05.

^a^One patient did not complete the food frequency questionnaire and was excluded from analysis.

^b^*p*-value obtained by Kruskal–Wallis test after pairwise comparison of patients with chronic pancreatitis to healthy controls after correction for multiple testing.

^c^*p*-value obtained by Kruskal–Wallis test after pairwise comparison of patients with liver cirrhosis to healthy controls after correction for multiple testing.

^d^*p*-value obtained by Kruskal–Wallis test after pairwise comparison of patients with chronic pancreatitis to liver cirrhosis after correction for multiple testing.

^e^Calculation is based on ideal body weight in subjects with ascites, edema, or obesity.

We found even more pronounced differences in micronutrient intake between patients and healthy subjects (Table 3). Patients with CP had higher intake of vitamin A whereas vitamin C consumption was lower than in controls. Lower quantities of vitamin C were also observed in patients with LC compared to healthy subjects. Moreover, patients with LC had lower intake of magnesium, iron and zinc as well as vitamin E and B6. Also regarding intake of micronutrients there were sex-specific differences (Supplementary Table 7). Reduced intake of vitamin C was only seen in male patients with CP or LC compared to healthy controls. Likewise, the increased consumption of vitamin A in CP as well as the decreased ingestion of magnesium and iron in LC was also exclusively observed in male patients. Conversely, only female patients with CP had a significantly decreased intake of zinc and folic acid compared to controls. On the other hand, female patients with LC reported a lower intake of vitamin B1 whereas in male patients there were no differences compared to healthy subjects.

**Table 3:** Micronutrient intake in patients with chronic pancreatitis or liver cirrhosis and healthy controls.

	Chronic pancreatitis (n = 65)^a^	Liver cirrhosis (n = 78)	Healthy controls (n = 94)	*p*-value^b^	*p*-value^c^	*p*-value^d^
Sodium, mg/d	2309 (1177)	1859 (1209)	2132 (1085)	1.000	0.266	0.058
Potassium, mg/d	2869 (1451)	2819 (1705)	3154 (1682)	0.437	0.091	1.000
Calcium, mg/d	890 (703)	860 (550)	1032 (735)	0.481	0.094	1.000
Phosphorus, mg/d	1322 (670)	1217 (798)	1422 (706)	1.000	0.195	1.000
Magnesium, mg/d	366 (161)	352 (162)	406 (312)	0.28	**0.001**	0.276
Iron, mg/d	15 (6)	13 (7)	15 (9)	0.605	**0.001**	0.142
Zinc, mg/d	12 (6)	12 (8)	13 (8)	0.232	**0.038**	1.000
Vitamin A, µg/d	1899 (3341)	1563 (2609)	1050 (1512)	**0.023**	0.095	1.000
Vitamin E, mg/d	9 (6)	8 (6)	10 (7)	0.165	**0.021**	1.000
Vitamin B1, mg/d	1 (1)	1 (1)	1 (1)	1.000	0.604	0.546
Vitamin B2, mg/d	10 (36)	10 (35)	18 (35)	1.000	0.418	0.807
Vitamin B6, mg/d	2 (1)	2 (1)	2 (1)	0.078	**0.047**	1.000
Folic acid, µg/d	214 (120)	223 (148)	259 (151)	0.074	0.192	1.000
Vitamin B12, µg/d	7 (7)	6 (6)	6 (5)	0.280	1.000	0.921
Vitamin C, mg/d	124 (134)	150 (153)	235 (219)	** < 0.001**	**0.003**	0.708

All data is presented as median (IQR); bold typed numbers indicate *p*-value < 0.05.

^a^One patient did not complete the food frequency questionnaire and was excluded from analysis.

^b^*p*-value obtained by Kruskal–Wallis test after pairwise comparison of patients with chronic pancreatitis to healthy controls after correction for multiple testing.

^c^*p*-value obtained by Kruskal–Wallis test after pairwise comparison of patients with liver cirrhosis to healthy controls after correction for multiple testing.

^d^*p*-value obtained by Kruskal–Wallis test after pairwise comparison of patients with chronic pancreatitis to liver cirrhosis after correction for multiple testing.

### Comparison of dietary quality and food group intake

**Figure 3:**
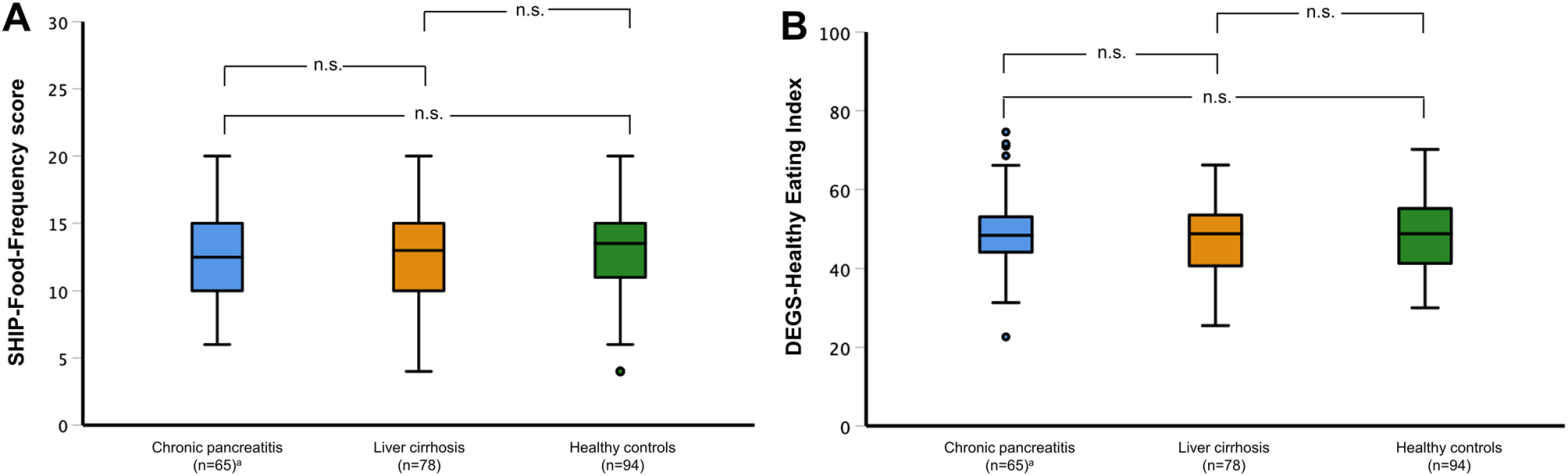
Comparison of dietary quality. Dietary quality in patients with chronic pancreatitis or liver cirrhosis and healthy controls measured by SHIP-Food-Frequency score (**A**) and DEGS-Healthy Eating Index (**B**). ^a^One patient did not complete the food frequency questionnaire and was excluded from analysis.

We next sought to investigate whether besides altered nutrient intake there were also differences in overall dietary patterns of patients with CP or LC compared to healthy individuals. We therefore studied dietary quality and food group consumption. Application of the SHIP-FFQ score and DEGS-HEI, as indicators of dietary quality, revealed no significant differences between both patient groups and healthy controls (Fig. 3). Sex-stratified analyses did not show any significant differences with regard to dietary quality either.

**Figure 4:**
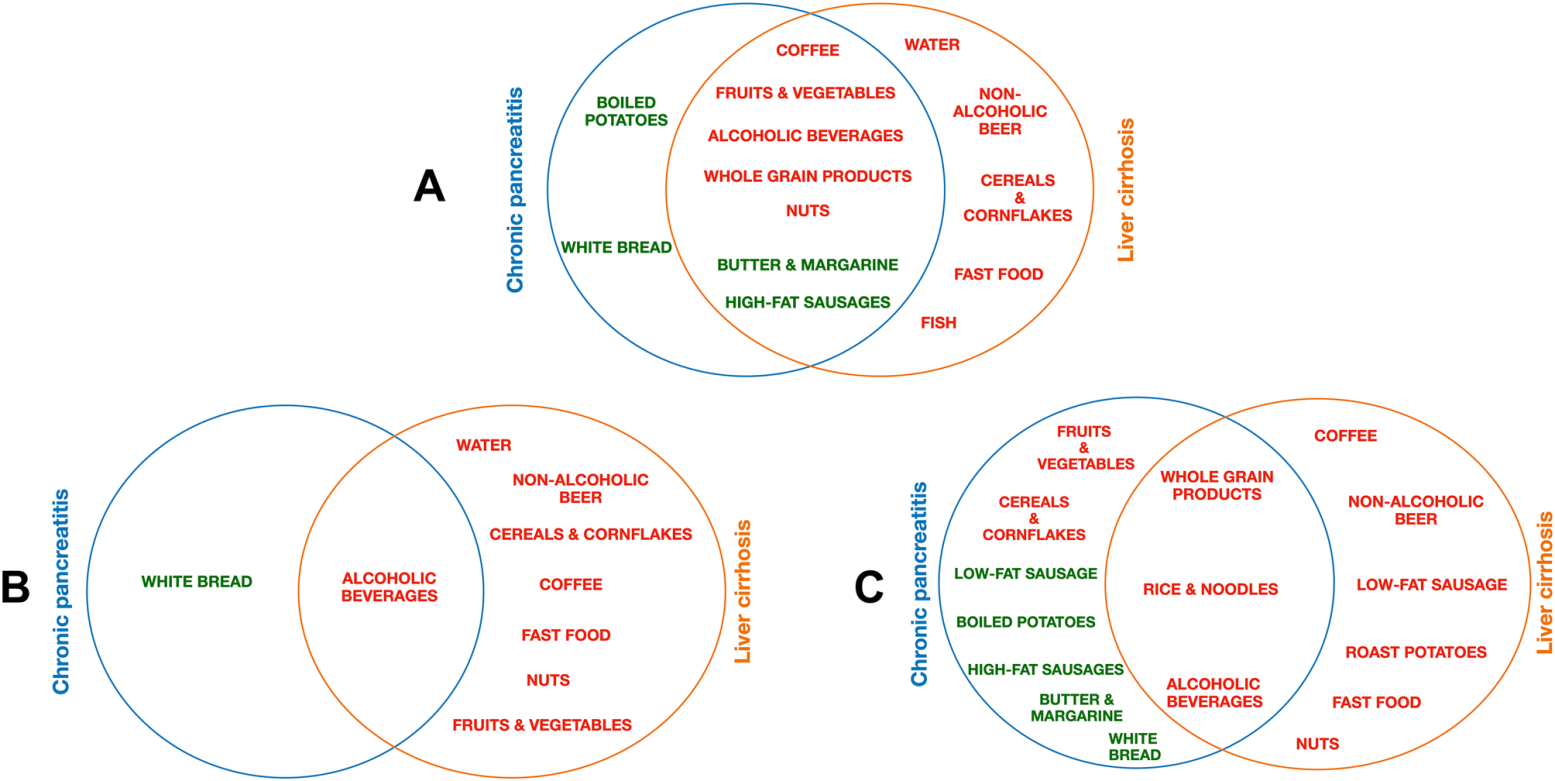
Comparison of food group intake. Differences in food group intake of patients with chronic pancreatitis or liver cirrhosis compared to healthy controls shown for all (**A**), female (**B**) and male subjects (**C**). Only food groups with *p*-value < 0.05 after correction for multiple testing are shown. Red color: reduced food consumption; green color: increased food consumption compared to healthy controls.

However, when comparing food group consumption, we found several significant differences, which were most pronounced between patients and healthy controls (Supplementary Tables 8, 9, 10). Results, including sex-stratified analyses, are graphically summarized in Fig. 4. In both patient groups, there was a decreased consumption of coffee, fruits & vegetables, whole grain products, nuts and alcoholic beverages compared to healthy controls whereas intake of butter and margarine and high-fat sausages was increased. In particular, boiled potatoes and white bread were preferred in CP patients, while patients with LC were characterized by reduced intakes of fast food, fish, cereals and cornflakes, water and non-alcoholic beer. The stratified subgroup analyses for male and female subjects revealed that the differences to healthy controls were largely influenced by sex. Alcoholic beverages were the only food group that was consistently less consumed in both male and female patients.

## Discussion

In this work, we comprehensively investigated the habitual dietary intake of patients with CP or LC to determine its potential contribution to impaired nutritional status in these chronic gastrointestinal diseases. Unexpectedly, in both diseases we did not find any fundamental differences between malnourished and non-malnourished patients regarding energy, nutrient or food group intake as well as overall dietary quality. However, comparison with healthy volunteers revealed that there were specific disease-related deviations in food and nutrient intake of both CP and LC patients.

The link between impaired nutritional status and dietary intake in CP and LC has been previously investigated in only a small number of studies^34–38^ and results have been inconclusive so far. Regarding CP, one study from India^34^ found lower caloric intake in patients with CP and weight loss > 10% after onset of disease compared to subjects with lower or no weight loss but no differences in macronutrient intake. Conversely, in another work, performed in India as well, there were no significant differences between patients with and without weight loss > 10% whereas a higher relative protein consumption was observed in those who lost weight^35^. A third trial reported a close correlation between lower BMI and decreased intake of energy and carbohydrates^36^. Likewise, findings in LC have been inconsistent so far. For instance, one study^37^ found lower energy intake and non-protein calorie to nitrogen ratio in patients with sarcopenia, a condition closely related to malnutrition. On the other hand, Riggio et al.^38^ demonstrated that in patients with stable LC, malnutrition defined by anthropometric measures was associated with altered relative intakes of carbohydrates and fat but not energy. In our study, after stratification for sex we found similar intakes of energy and macronutrients between malnourished and non-malnourished patients with CP or LC. Our findings, once more, highlight the importance of the GLIM consensus criteria in pathological conditions and support the underlying concept that besides reduced food intake, malassimilation and systemic inflammation present relevant etiologic factors contributing to the development of malnutrition in chronic gastrointestinal diseases as our previous works have suggested^22,23^.

Whereas dietary intake in patients with CP or LC was unrelated to nutritional status, we found it to be associated rather with disease itself. When we compared diets of patients with healthy volunteers, we observed several alterations in nutrient and food group consumption but not in energy intake or overall dietary quality. In agreement with our findings, a recent meta-analysis^39^ reported comparable energy intake between patients with CP and healthy controls. However, this analysis showed higher alcohol consumption, contributing to total energy intake, in patients with CP compared to controls, especially when disease was alcohol-related. Moreover, increased protein consumption was observed in patients in this meta-analysis while fat and carbohydrate intakes were similar to healthy controls. Likely, these divergent findings can be explained by the substantial heterogeneity of the studies included in the meta-analysis in terms of study design and methodology. In our own investigation, for instance, around two thirds of patients were teetotalers, which could explain the observed lower alcohol consumption than in controls. Moreover, higher protein consumption in patients seems to be linked to alcoholic CP as well, which might be the reason why we did not detect significant differences in our work. In patients with LC, besides lower alcohol consumption we found some further deviations from macronutrient intake of healthy volunteers. While energy intake was likewise similar, intake of dietary fiber, for instance, was lower than among controls in both male and female patients. Our findings regarding energy intake are supported by previous studies that found comparable energy intake between patients with LC and healthy controls^40–42^. By contrast, intake of dietary fiber in comparison with healthy controls has not been studied so far. It is therefore a highly relevant finding that we found lowered consumption in patients as benefits of higher dietary fiber intake, especially in patients with hepatic encephalopathy, have been highlighted in international guidelines^43,44^ and a recent work showed lower intake to be associated with shorter survival times^45^. Lower intake of dietary fiber in patients with LC could be both cause and effect of disease. Growing evidence suggests that among other factors low fiber intake could facilitate progression along the continuum of liver diseases via the gut-liver axis^46^. On the other hand, patients with LC often experience gastrointestinal symptoms, e.g. bloating,^47^ which might be amplified when consuming fiber-rich foods. Although our study does not allow any conclusions regarding causal relations, our findings underline the need for future investigations on this aspect.

As opposed to energy and macronutrients, alterations in micronutrient consumption were more abundant in both patient groups. In CP and LC patients, altered intake can be linked to the differences in food group consumption that we found in comparison to healthy controls. The higher intake of vitamin A in patients with CP was due to higher quantities of fat-rich sausages and spreads, the most relevant food groups in terms of vitamin A consumption, while lesser ingestion of vitamin C resulted from the lower amounts of fruits and vegetables consumed. The latter can be equally concluded for patients with LC but here we found even greater differences. Micronutrient intake in LC patients was characterized by lower quantities of magnesium, iron, zinc, vitamin B6 and E, which can be attributed to the reduced consumption of nuts and whole-grain products. Although these food groups were also eaten less than in controls by patients with CP, higher intakes of boiled potatoes, fat-rich sausages and spreads likely counterbalanced ingestion of these nutrients. Altered micronutrient intake has been previously described in cohorts of both CP^36,48^ and LC patients^43,49^. However, not all of these studies used a controlled design and findings have been inconsistent, which suggests that differences likely depend on the respective study population, i.e. findings might vary between different etiologies or geographical regions. Acknowledging these discrepancies and the fact that nutrient deficiency is not defined by reduced intake, the clinical implications of our findings regarding altered micronutrient intake require further investigation.

In this respect, it is noteworthy that overall dietary quality in patients was comparable to controls despite the disease-specific alterations in nutrient and food group intake that we found. Previous studies reported less healthy dietary habits in both CP^48^ and LC^42,50^, which suggested that patients may benefit from improving quality of dietary intake in terms of nutritional status and disease progression. However, because of methodological issues these earlier studies might be biased. For instance, in a study investigating dietary quality in patients with CP^48^, the control group was matched for age but not sex and thus included significantly more females. A generally higher quality of diet in women than in men has been shown repeatedly and across various geographical regions^51,52^ and would explain lower dietary quality in predominantly male cohorts of CP patients. As for LC, one study compared quality of diet in patients to the general public without matching for age or sex^50^ and in another trial diet quality was judged rather subjectively without employing any aggregated score^42^. By contrast, in our own work we applied two validated indices of dietary quality and accounted for differences in sex distributions. Moreover, in our analyses we also observed some healthier traits in patients. Besides less alcohol consumption, we also found higher consumption of boiled potatoes in CP and less fast-food in LC patients, which supports our finding that dietary quality is not generally reduced in patients with CP or LC.

## Limitations

There are some limitations to our work which should be acknowledged. First, because of the cross-sectional study design it is not possible to entirely dismiss a causal link between habitual dietary intake and malnutrition. Dietary habits of patients may have changed due to the disease or deteriorated nutritional status. However, studies have shown that habitual diet in general is largely stable for at least 5 years^53,54^ and many patients with CP or LC fail to adhere to disease-specific dietary recommendations^55,56^. Moreover, in CP and LC other mechanisms such as an increased resting energy expenditure due to systemic inflammation or malabsorption can contribute to impaired nutritional status. Thus, it is plausible that malnutrition not necessarily results from reduced dietary intake and only a longitudinally designed studied could definitely answer this question. Secondly, we cannot entirely rule out selection and social desirability bias, which could result in higher rates of alcohol abstinence and more favorable dietary behavior among patients. Yet, the fact that patients also reported several unhealthier lifestyle habits, e.g. smoking or lower consumption of fruits and vegetables, questions this possibility. Nevertheless, external validity may be limited in cohorts with divergent clinical and patient characteristics. Last, it must be considered that dietary assessment is challenging as there is no objective method to comprehensively measure habitual dietary intake. In particular, FFQs have been criticized as both over- and underreporting of dietary intake may occur. However, we used two instruments that have shown sufficient relative validity before. Moreover, all subjects answered identical FFQs and we focused our analyses on relative differences between groups rather than absolute numbers. Hence, it is unlikely that our results are biased by the methodological limitations of dietary assessment.

## Conclusion

We show that habitual dietary intake of patients with CP or LC is rather related to disease than to nutritional status, which supports that in both entities malassimilation and systemic inflammation relevantly contribute to the development of malnutrition. The observation that patients show favorable dietary behavior such as alcohol abstinence as well as comparable overall dietary quality suggests that there is dietary awareness in the majority of subjects. This highlights the importance and potential of offering targeted nutrition therapy to patients with CP or LC, beginning with dietary counseling.

### Supplementary Information

Supplementary Table S1.

Supplementary Table S2.

Supplementary Table S3.

Supplementary Table S4.

Supplementary Table S5.

Supplementary Table S6.

Supplementary Table S7.

Supplementary Table S8.

Supplementary Table S9.

Supplementary Table S10.

## Data Availability

The datasets used and/or analyzed during the current study are available from the corresponding author on reasonable request.

## Abbreviations

BIA: Bioelectrical impedance analysis
BMI: Body mass index
COPPS: Chronic Pancreatitis Prognosis Score
CP: Chronic pancreatitis
DEGS-HEI: DEGS-Healthy Eating Index
FFQ: Food frequency questionnaire
GLIM: Global Leadership Initiative on Malnutrition
LC: Liver cirrhosis
NRS-2002: Nutritional Risk Screening 2002
