# Supplementary material for: Nutritional status in patients with chronic pancreatitis and liver cirrhosis is related to disease conditions and not dietary habits
Source: Sci Rep. 2024 Feb 26;14:4700. doi: 10.1038/s41598-024-54998-7 (PMC10897307; doi:10.1038/s41598-024-54998-7)
Supplement: Supplementary file 1 — Supplementary Table S1. [file 41598_2024_54998_MOESM1_ESM.docx]

**Supplementary Table 1:** Comparison of energy, macro- and micronutrient intake in patients with chronic pancreatitis or liver cirrhosis with and without malnutrition

|  | **Chronic pancreatitis**  **with malnutrition (n=42)** | **Chronic pancreatitis without malnutrition**  **(n=23)^a^** | **Liver cirrhosis with**  **malnutrition (n=48)** | **Liver cirrhosis without malnutrition (n=30)** | **p-value^b^** | **p-value^c^** |
| --- | --- | --- | --- | --- | --- | --- |
| **Macronutrients** |  |  |  |  |  |  |
| Energy, kcal/d | 2114 (1055) | 1576 (1421) | 1908 (1420) | 1886 (887) | **0.031** | 0.902 |
| Energy, kcal/kg body weight^d^/d | 30.0 (18.3) | 25.3 (13.6) | 28.2 (16.4) | 28.1 (14.4) | **0.038** | 0.659 |
| Protein, g/d | 80 (34) | 59 (42) | 65 (53) | 68 (36) | 0.162 | 0.955 |
| Protein, g/kg body weight^d^/d | 1.2 (0.5) | 1.0 (0.5) | 1.0 (0.7) | 1.0 (0.6) | 0.105 | 0.742 |
| Fat, g/d | 80 (46) | 55 (61) | 58 (65) | 57 (50) | 0.074 | 0.873 |
| Saturated fatty acids, g/d | 42 (27) | 31 (39) | 30 (33) | 27 (21) | 0.136 | 0.511 |
| Monounsaturated fatty acids, g/d | 28 (14) | 23 (27) | 20 (20) | 20 (21) | 0.163 | 0.813 |
| Polyunsaturated fatty acids, g/d | 10 (7) | 7 (9) | 8 (10) | 8 (7) | 0.173 | 0.967 |
| Cholesterol, g/d | 309 (227) | 232 (199) | 252 (230) | 260 (265) | 0.188 | 0.655 |
| Carbohydrates, g/d | 250 (125) | 181 (136) | 196 (162) | 225 (111) | **0.025** | 0.971 |
| Alcohol, g/d | 0 (3.5) | 0 (4) | 0 (1.5) | 1 (27) | 0.601 | 0.060 |
| Dietary fiber, g/d | 22 (13) | 21 (18) | 19 (17) | 18 (13) | 0.826 | 0.988 |
| Water, ml/d | 2720 (1786) | 2904 (1943) | 2599 (1517) | 2745 (1294) | 0.940 | 0.551 |
| **Micronutrients** |  |  |  |  |  |  |
| Sodium, mg/d | 2390 (1124) | 2059 (1561) | 1707 (1379) | 2133 (1318) | 0.225 | 0.479 |
| Potassium, mg/d | 3047 (1098) | 2651 (1831) | 2882 (2080) | 2724 (1364) | 0.190 | 0.845 |
| Calcium, mg/d | 963 (664) | 797 (674) | 968 (667) | 675 (439) | 0.291 | 0.162 |
| Phosphorus, mg/d | 1397 (556) | 1047 (697) | 1225 (996) | 1168 (679) | 0.105 | 0.922 |
| Magnesium, mg/d | 372 (133) | 344 (253) | 332 (167) | 378 (170) | 0.269 | 0.327 |
| Iron, mg/d | 15 (5) | 13 (9) | 12 (7) | 14 (6) | 0.349 | 0.212 |
| Zinc, mg/d | 12 (4) | 11 (9) | 12 (9) | 12 (8) | 0.292 | 0.841 |
| Vitamin A, µg/d | 1914 (3089) | 1393 (4965) | 1391 (2344) | 1728 (3097) | 0.471 | 0.435 |
| Vitamin E, mg/d | 10 (5) | 8 (7) | 8 (7) | 8 (3) | 0.352 | 0.836 |
| Vitamin B1, mg/d | 2 (1) | 1 (1) | 1 (1) | 1 (1) | 0.188 | 0.592 |
| Vitamin B2, mg/d | 10 (36) | 15 (54) | 14 (35) | 5 (36) | 0.549 | 0.335 |
| Vitamin B6, mg/d | 2 (1) | 1 (1) | 2 (1) | 2 (1) | 0.417 | 0.921 |
| Folic acid, µg/d | 220 (140) | 199 (135) | 223 (168) | 232 (140) | 1.000 | 0.746 |
| Vitamin B12, µg/d | 7 (5) | 7 (10) | 6 (5) | 6 (8) | 0.591 | 0.657 |
| Vitamin C, mg/d | 118 (138) | 147 (118) | 150 (171) | 147 (138) | 0.569 | 0.482 |

All data is presented as median (IQR); bold type numbers indicate p-value < 0.05

^a^ one patient did not complete the food frequency questionnaire and was excluded from analysis

^b^ p-value obtained from Mann-Whitney U test after pairwise comparison between patients with chronic pancreatitis with and without malnutrition

^c^ p-value obtained from Mann-Whitney U test after pairwise comparison between patients with liver cirrhosis with and without malnutrition

^d^ Calculation is based on ideal body weight in subjects with ascites, edema, or obesity
