# Supplementary material for: Nutritional status in patients with chronic pancreatitis and liver cirrhosis is related to disease conditions and not dietary habits
Source: Sci Rep. 2024 Feb 26;14:4700. doi: 10.1038/s41598-024-54998-7 (PMC10897307; doi:10.1038/s41598-024-54998-7)
Supplement: Supplementary file 2 — Supplementary Table S2. [file 41598_2024_54998_MOESM2_ESM.docx]

**Supplementary Table 2:** Comparison of food group consumption in patients with chronic pancreatitis or liver cirrhosis with and without malnutrition

|  | **Chronic pancreatitis**  **with malnutrition (n=42)** | **Chronic pancreatitis without malnutrition**  **(n=23)^a^** | **Liver cirrhosis with**  **malnutrition (n=48)** | **Liver cirrhosis without malnutrition (n=30)** | **p-value^b^** | **p-value^c^** |
| --- | --- | --- | --- | --- | --- | --- |
| Water, ml/d | 1050 (1200) | 1200 (2000) | 700 (600) | 900 (600) | 0.968 | 0.174 |
| Light drinks, ml/d | 0 (0) | 0 (7) | 0 (0) | 0 (0) | 0.199 | 0.292 |
| Lemonade, ml/d | 59 (192) | 9 (43) | 164 (447) | 143 (370) | **0.027** | 0.486 |
| Coffee, ml/d | 150 (439) | 450 (600) | 75 (137) | 38 (300) | 0.169 | 0.975 |
| Tea, ml/d | 241 (346) | 32 (300) | 150 (268) | 150 (228) | **0.013** | 0.778 |
| Alcoholic beverages, ml/d | 0 (29) | 0 (75) | 0 (67) | 0 (81) | 0.962 | 0.848 |
| Beer, ml/d | 0 (15) | 0 (71) | 0 (0) | 0 (36) | 0.759 | 0.447 |
| Non-alcoholic beer, ml/d | 0 (29) | 0 (12) | 0 (0) | 0 (0) | 0.453 | 0.058 |
| Wine & sparkling wine, ml/d | 0 (4) | 0 (0) | 0 (0) | 0 (0) | 0.057 | 0.458 |
| High-percentage alcoholic drinks, ml/d | 0 (0) | 0 (0) | 0 (0) | 0 (0) | 0.749 | 0.181 |
| Cocktails, ml/d | 0 (0) | 0 (0) | 0 (0) | 0 (0) | 0.212 | 0.875 |
| White bread, g/d | 121 (148) | 52 (112) | 43 (88) | 92 (83) | **0.006** | 0.107 |
| Whole grain products, g/d | 7 (50) | 21 (50) | 11 (50) | 12 (113) | 0.445 | 0.297 |
| Cereals & cornflakes, g/d | 0 (5) | 0 (1) | 0 (0) | 0 (4) | 0.968 | 0.135 |
| Fruits & vegetables, g/d | 202 (240) | 295 (337) | 231 (356) | 359 (330) | 0.266 | 0.213 |
| Rice & noodles, g/d | 17 (16) | 25 (29) | 21 (41) | 13 (40) | 0.430 | 0.415 |
| Boiled potatoes, g/d | 88 (109) | 88 (50) | 38 (69) | 88 (105) | **0.041** | 0.400 |
| Roast potatoes, g/d | 5 (12) | 0 (13) | 0 (7) | 5 (7) | 0.092 | 0.417 |
| Low-fat dairy products, g/d | 0 (0) | 0 (0) | 0 (0) | 0 (0) | 1.000 | 1.000 |
| Dairy products, g/d | 110 (165) | 102 (153) | 127 (197) | 170 (203) | 0.915 | 0.535 |
| Eggs, g/d | 13 (13) | 13 (15) | 13 (21) | 26 (19) | 0.251 | **0.008** |
| Low-fat sausages, g/d | 4 (8) | 2 (10) | 0 (4) | 2 (9) | 0.690 | 0.163 |
| High-fat sausages, g/d | 20 (36) | 13 (59) | 10 (24) | 17 (31) | 0.712 | 0.063 |
| Meat & poultry, g/d | 46 (41) | 46 (76) | 32 (44) | 64 (66) | 0.678 | **0.017** |
| Fish, g/d | 13 (17) | 12 (15) | 3 (19) | 11 (22) | 0.625 | 0.106 |
| Butter & margarine, g/d | 15 (13) | 10 (17) | 9 (17) | 10 (15) | 0.094 | 0.170 |
| Fast Food, g/d | 16 (29) | 15 (30) | 5 (27) | 5 (24) | 0.449 | 0.690 |
| Crisps, salty pastries, crackers, g/d | 0 (2) | 0 (1) | 0 (6) | 0 (0) | 0.575 | 0.098 |
| Desserts & sweet spreads, g/d | 122 (110) | 32 (106) | 52 (97) | 70 (113) | **0.017** | 0.160 |
| Nuts, g/d | 0 (4) | 0 (5) | 0 (0) | 0 (1) | 0.971 | 0.590 |

All data is presented as median (IQR); bold typed numbers indicate p-value < 0.05

^a^ one patient did not complete the food frequency questionnaire and was excluded from analysis

^b^ p-value obtained from Mann-Whitney U test after pairwise comparison between patients with chronic pancreatitis with and without malnutrition

^c^ p-value obtained from Mann-Whitney U test after pairwise comparison between patients with liver cirrhosis with and without malnutrition
