# Supplementary material for: Nutritional status in patients with chronic pancreatitis and liver cirrhosis is related to disease conditions and not dietary habits
Source: Sci Rep. 2024 Feb 26;14:4700. doi: 10.1038/s41598-024-54998-7 (PMC10897307; doi:10.1038/s41598-024-54998-7)
Supplement: Supplementary file 3 — Supplementary Table S3. [file 41598_2024_54998_MOESM3_ESM.docx]

**Supplementary Table 3:** Comparison of food group consumption in female patients with chronic pancreatitis or liver cirrhosis with and without malnutrition

|  | **Chronic pancreatitis with malnutrition**  **(n=7)** | **Chronic pancreatitis without malnutrition**  **(n=8)** | **Liver cirrhosis with malnutrition**  **(n=17)** | **Liver cirrhosis without malnutrition**  **(n=9)** | **p-value^a^** | **p-value^b^** |
| --- | --- | --- | --- | --- | --- | --- |
| Water, ml/d | 900 (600) | 1500 (2738) | 1200 (600) | 900 (700) | 0.681 | 0.842 |
| Light drinks, ml/d | 0 (0) | 0 (7) | 0 (0) | 0 (4) | 0.470 | 0.628 |
| Lemonade, ml/d | 43 (104) | 2 (21) | 100 (295) | 200 (290) | 0.299 | 0.798 |
| Coffee, ml/d | 300 (332) | 177 (656) | 150 (278) | 150 (219) | 0.536 | 0.511 |
| Tea, ml/d | 332 (889) | 375 (474) | 182 (243) | 139 (193) | 0.837 | 0.315 |
| Alcoholic beverages, ml/d | 0 (19) | 0 (0) | 0 (11) | 0 (6) | 0.252 | 0.887 |
| Beer, ml/d | 0 (6) | 0 (0) | 0 (0) | 0 (0) | 0.536 | 0.669 |
| Non-alcoholic beer, ml/d | 0 (15) | 0 (9) | 0 (0) | 0 (0) | 0.837 | 0.842 |
| Wine & sparkling wine, ml/d | 0 (11) | 0 (0) | 0 (0) | 0 (6) | 0.174 | 0.588 |
| High-percentage alcoholic drinks, ml/d | 0 (1) | 0 (0) | 0 (0) | 0 (0) | 0.351 | 0.511 |
| Cocktails, ml/d | 0 (0) | 0 (0) | 0 (0) | 0 (0) | 1.000 | 1.000 |
| White bread, g/d | 51 (154) | 30 (46) | 25 (67) | 46 (84) | 0.114 | 0.549 |
| Whole grain products, g/d | 25 (49) | 21 (47) | 21 (67) | 0 (81.5) | 0.536 | 0.262 |
| Cereals & cornflakes, g/d | 0 (25) | 4 (11) | 0 (0) | 0 (7) | 0.918 | 0.215 |
| Fruits & vegetables, g/d | 188 (374) | 430 (361) | 210 (194) | 332 (429) | 0.210 | 0.406 |
| Rice & noodles, g/d | 14 (36) | 35 (54) | 12 (40) | 9 (47) | 0.299 | 0.628 |
| Boiled potatoes, g/d | 138 (100) | 38 (104) | 38 (71) | 88 (135) | 0.252 | 0.315 |
| Roast potatoes, g/d | 5 (4) | 0 (10) | 0 (5) | 5 (5) | 0.252 | 0.440 |
| Low-fat dairy products, g/d | 0 (0) | 0 (0) | 0 (0) | 0 (0) | 1.000 | 1.000 |
| Dairy products, g/d | 164 (198) | 78 (87) | 131 (207) | 208 (380) | 0.252 | 0.194 |
| Eggs, g/d | 13 (21) | 13 (16) | 13 (23) | 13 (7) | 0.758 | 0.669 |
| Low-fat sausages, g/d | 1 (2) | 0 (2) | 0 (9) | 1 (9) | 1.000 | 0.798 |
| High-fat sausages, g/d | 4 (20) | 1 (1) | 10 (17) | 17 (27) | 0.299 | 0.588 |
| Meat & poultry, g/d | 38 (25) | 45 (74) | 19 (36) | 64 (53) | 0.470 | 0.110 |
| Fish, g/d | 16 (25) | 18 (20) | 5 (21) | 10 (28) | 0.837 | 0.669 |
| Butter & margarine, g/d | 10 (30) | 4 (6) | 5 (11) | 10 (17) | **0.012** | 0.315 |
| Fast Food, g/d | 9 (8) | 8 (22) | 5 (27) | 5 (19) | 1.000 | 0.475 |
| Crisps, salty pastries, crackers, g/d | 0 (3) | 1 (2) | 0 (6) | 0 (0) | 1.000 | 0.194 |
| Desserts & sweet spreads, g/d | 122 (99) | 47 (63) | 41 (144) | 120 (108) | 0.408 | 0.588 |
| Nuts, g/d | 1 (3) | 1 (3) | 0 (1) | 0 (3) | 0.837 | 0.754 |

All data is presented as median (IQR); bold typed numbers indicate p-value < 0.05

^a^ p-value obtained from Mann-Whitney U test after pairwise comparison between patients with chronic pancreatitis with and without malnutrition

^b^ p-value obtained from Mann-Whitney U test after pairwise comparison between patients with liver cirrhosis with and without malnutrition
