# Supplementary material for: Nutritional status in patients with chronic pancreatitis and liver cirrhosis is related to disease conditions and not dietary habits
Source: Sci Rep. 2024 Feb 26;14:4700. doi: 10.1038/s41598-024-54998-7 (PMC10897307; doi:10.1038/s41598-024-54998-7)
Supplement: Supplementary file 4 — Supplementary Table S4. [file 41598_2024_54998_MOESM4_ESM.docx]

**Supplementary Table 4:** Comparison of food group consumption in male patients with chronic pancreatitis or liver cirrhosis with and without malnutrition

|  | **Chronic pancreatitis with malnutrition**  **(n=35)** | **Chronic pancreatitis without malnutrition**  **(n=15)^a^** | **Liver cirrhosis with malnutrition**  **(n=31)** | **Liver cirrhosis without malnutrition**  **(n=21)** | **p-value^b^** | **p-value^c^** |
| --- | --- | --- | --- | --- | --- | --- |
| Water, ml/d | 1200 (1200) | 900 (2086) | 600 (750) | 900 (700) | 0.662 | 0.067 |
| Light drinks, ml/d | 0 (0) | 0 (7) | 0 (0) | 0 (0) | 0.319 | 0.368 |
| Lemonade, ml/d | 64 (382) | 18 (207) | 200 (593) | 86 (407) | 0.136 | 0.460 |
| Coffee, ml/d | 150 (445) | 450 (364) | 32 (150) | 38 (300) | **0.041** | 0.616 |
| Tea, ml/d | 182 (300) | 5 (64) | 150 (300) | 150 (196) | **<0.001** | 0.262 |
| Alcoholic beverages, ml/d | 0 (42) | 0 (211) | 0 (480) | 0 (277) | 0.295 | 0.798 |
| Beer, ml/d | 0 (24) | 0 (141) | 0 (0) | 0 (201) | 0.247 | 0.345 |
| Non-alcoholic beer, ml/d | 0 (29) | 0 (12) | 0 (0) | 0 (6) | 0.601 | **0.028** |
| Wine & sparkling wine, ml/d | 0 (4) | 0 (0) | 0 (4) | 0 (0) | 0.342 | 0.155 |
| High-percentage alcoholic drinks, ml/d | 0 (0) | 0 (4) | 0 (1) | 0 (0) | 0.196 | 0.334 |
| Cocktails, ml/d | 0 (0) | 0 (0) | 0 (0) | 0 (0) | 0.352 | 0.800 |
| White bread, g/d | 121 (129) | 71 (108) | 50 (85) | 96 (97) | 0.077 | 0.228 |
| Whole grain products, g/d | 4 (50) | 21 (96) | 4 (39) | 25 (148) | 0.180 | **0.037** |
| Cereals & cornflakes, g/d | 0 (2) | 0 (0) | 0 (0) | 0 (3) | 0.235 | 0.608 |
| Fruits & vegetables, g/d | 225 (207) | 214 (329) | 241 (416) | 359 (300) | 0.966 | 0.461 |
| Rice & noodles, g/d | 17 (16) | 17 (24) | 27 (45) | 13 (40) | 0.882 | 0.424 |
| Boiled potatoes, g/d | 88 (137) | 88 (50) | 75 (119) | 88 (85) | 0.141 | 0.895 |
| Roast potatoes, g/d | 5 (13) | 0 (13) | 0 (13) | 0 (8) | 0.236 | 0.709 |
| Low-fat dairy products, g/d | 0 (0) | 0 (0) | 0 (0) | 0 (0) | 1.000 | 1.000 |
| Dairy products, g/d | 106 (155) | 110 (215) | 116 (209) | 136 (185) | 0.452 | 0.970 |
| Eggs, g/d | 26 (13) | 13 (15) | 13 (21) | 26 (47) | 0.323 | **0.007** |
| Low-fat sausages, g/d | 4 (9) | 4 (19) | 0 (4) | 2 (7) | 0.765 | 0.143 |
| High-fat sausages, g/d | 20 (31) | 40 (67) | 9 (36) | 17 (31) | 0.154 | 0.073 |
| Meat & poultry, g/d | 46 (55) | 75 (80) | 35 (59) | 64 (72) | 0.710 | 0.117 |
| Fish, g/d | 11 (17) | 11 (9) | 3 (19) | 12 (22) | 0.476 | 0.125 |
| Butter & margarine, g/d | 15 (15) | 15 (25) | 10 (15) | 10 (15) | 0.983 | 0.505 |
| Fast Food, g/d | 17 (33) | 16 (67) | 4 (33) | 6 (30) | 0.640 | 0.977 |
| Crisps, salty pastries, crackers, g/d | 0 (2) | 0 (0) | 0 (8) | 0 (2) | 0.312 | 0.314 |
| Desserts & sweet spreads, g/d | 122 (108) | 27 (156) | 54 (95) | 68 (119) | **0.041** | 0.201 |
| Nuts, g/d | 0 (5) | 0 (5) | 0 (0) | 0 (1) | 0.835 | 0.401 |

All data is presented as median (IQR); bold typed numbers indicate p-value < 0.05

^a^ one patient did not complete the food frequency questionnaire and was excluded from analysis

^b^ p-value obtained from Mann-Whitney U test after pairwise comparison between patients with chronic pancreatitis with and without malnutrition

^c^ p-value obtained from Mann-Whitney U test after pairwise comparison between patients with liver cirrhosis with and without malnutrition
