# Supplementary material for: Nutritional status in patients with chronic pancreatitis and liver cirrhosis is related to disease conditions and not dietary habits
Source: Sci Rep. 2024 Feb 26;14:4700. doi: 10.1038/s41598-024-54998-7 (PMC10897307; doi:10.1038/s41598-024-54998-7)
Supplement: Supplementary file 5 — Supplementary Table S5. [file 41598_2024_54998_MOESM5_ESM.docx]

**Supplementary Table 5:** Comparison of dietary quality in patients with chronic pancreatitis or liver cirrhosis with and without malnutrition by SHIP-Food-Frequency score and DEGS-Healthy Eating Index unstratified and stratified by sex

|  | **Chronic pancreatitis with malnutrition** | **Chronic pancreatitis without malnutrition** | **Liver cirrhosis with malnutrition** | **Liver cirrhosis without malnutrition** | **p-value^b^** | **p-value^c^** |
| --- | --- | --- | --- | --- | --- | --- |
| **Total** | **(n=42)** | **(n=23)^a^** | **(n=48)** | **(n=30)** |  |  |
| SHIP-Food-Frequency score | 12.15 (±3.37) | 13.04 (±2.65) | 11.91 (±3.12) | 13.43 (±3.45) | 0.264 | **0.049** |
| DEGS-Healthy Eating Index | 49.45 (±9.83) | 48.28 (±9.18) | 47.18 (±9.60) | 47.05 (±11.18) | 0.635 | 0.957 |
| **Female** | **(n=7)** | **(n=8)** | **(n=17)** | **(n=9)** |  |  |
| SHIP-Food-Frequency score | 12.71 (±4.19) | 14.00 (±2.73) | 12.59 (±2.48) | 14.11 (±2.15) | 0.488 | 0.132 |
| DEGS-Healthy Eating Index | 49.59 (±11.27) | 55.07 (±11.19) | 49.55 (±8.27) | 47.18 (±10.87) | 0.362 | 0.540 |
| **Male** | **(n=35)** | **(n=15)** | **(n=31)** | **(n=21)** |  |  |
| SHIP-Food-Frequency score | 12.00 (±6.00) | 12.5 (±3.00) | 11.53 (±3.41) | 13.41 (±3.89) | 0.539 | 0.124 |
| DEGS-Healthy Eating Index | 49.43 (±9.69) | 44.87 (±5.82) | 45.88 (±10.15) | 46.99 (±11.58) | 0.090 | 0.716 |

All data is presented as mean (±SD); bold typed numbers indicate p-value < 0.05
^a^ one patient did not complete the food frequency questionnaire and was excluded from analysis

^b^ p-value obtained by t-test comparing patients with chronic pancreatitis with and without malnutrition
^c^ p-value obtained by t-test comparing patients with liver cirrhosis with and without malnutrition
