# Supplementary material for: Nutritional status in patients with chronic pancreatitis and liver cirrhosis is related to disease conditions and not dietary habits
Source: Sci Rep. 2024 Feb 26;14:4700. doi: 10.1038/s41598-024-54998-7 (PMC10897307; doi:10.1038/s41598-024-54998-7)
Supplement: Supplementary file 6 — Supplementary Table S6. [file 41598_2024_54998_MOESM6_ESM.docx]

**Supplementary Table 6:** Comparison of energy and macronutrient intake in patients with chronic pancreatitis or liver cirrhosis and healthy controls stratified by sex

|  | **Female** | | | | | |  | **Male** | | | | | | |
| --- | --- | --- | --- | --- | --- | --- | --- | --- | --- | --- | --- | --- | --- | --- |
|  | **Chronic pancreatitis**  **(n=15)** | **Liver cirrhosis**  **(n=26)** | **Healthy controls**  **(n=49)** | **p-value^b^** | **p-value^c^** | **p-value^d^** |  | **Chronic pancreatitis (n=50)^a^** | | **Liver cirrhosis**  **(n=52)** | **Healthy controls**  **(n=45)** | **p-value^b^** | **p-value^c^** | **p-value**^d^ |
| Energy, kcal/d | 1223 (675) | 1674 (1046) | 1667 (554) | 0.154 | 1.000 | 0.474 |  | | 2114 (1101) | 2033 (1392) | 2112 (1134) | 1.000 | 1.000 | 1.000 |
| Energy, kcal/kg body weight^e^/d | 20.4 (10.4) | 27.5 (11.3) | 27.0 (11.2) | 0.352 | 1.000 | 0.448 |  | | 28.5 (17.5) | 28.6 (17.2) | 27.7 (15.0) | 1.000 | 1.000 | 1.000 |
| Protein, g/d | 55 (26) | 57 (45) | 69 (29) | 0.227 | 0.204 | 1.000 |  | | 82 (37) | 74 (48) | 87 (43) | 1.000 | 0.179 | 0.425 |
| Protein, g/kg body weight^e^/d | 0.9 (0.5) | 0.9 (0.8) | 1.0 (0.6) | 0.668 | 0.442 | 1.000 |  | | 1.1 (0.5) | 1.0 (0.7) | 1.1 (0.6) | 1.000 | 1.000 | 0.597 |
| Fat, g/d | 37 (31) | 49 (48) | 57 (25) | 0.216 | 1.000 | 0.749 |  | | 82 (44) | 59 (60) | 75 (36) | 1.000 | 1.000 | 0.278 |
| Saturated fatty acids, g/d | 18 (23) | 27 (21) | 27 (16) | 0.512 | 1.000 | 0.636 |  | | 42 (29) | 31 (31) | 38 (20) | 1.000 | 1.000 | 0.207 |
| Monounsaturated fatty acids, g/d | 13 (11) | 18 (16) | 20 (11) | 0.166 | 1.000 | 0.468 |  | | 29 (14) | 20 (23) | 27 (14) | 1.000 | 0.897 | 1.151 |
| Polyunsaturated fatty acids, g/d | 6 (4) | 7 (10) | 9 (6) | 0.074 | 0.312 | 1.000 |  | | 11 (7) | 9 (10) | 12 (7) | 1.000 | **0.043** | 0.137 |
| Cholesterol, g/d | 203 (73) | 214 (190) | 254 (139) | 0.380 | 0.584 | 1.000 |  | | 333 (239) | 275 (263) | 318 (141) | 1.000 | 0.935 | 0.484 |
| Carbohydrates, g/d | 146 (130) | 185 (127) | 178 (93) | 0.964 | 1.000 | 1.000 |  | | 243 (132) | 229 (151) | 227 (165) | 1.000 | 1.000 | 1.000 |
| Alcohol, g/d | 0 (0) | 0 (1.25) | 5 (9) | **< 0.001** | **< 0.001** | 1.000 |  | | 0 (6) | 0 (24) | 7 (12) | **0.002** | **0.006** | 1.000 |
| Dietary fiber, g/d | 21 (17) | 18 (20) | 23 (15) | 0.723 | **0.029** | 1.000 |  | | 23 (15) | 19 (16) | 28 (19) | 0.088 | **0.011** | 1.000 |
| Water, ml/d | 2732 (2808) | 2645 (1335) | 3253 (3977) | 0.475 | 0.079 | 1.000 |  | | 2794 (1777) | 2692 (1522) | 2974 (2969) | 1.000 | 0.541 | 0.445 |

All data is presented as median (IQR); bold typed numbers indicate p-value < 0.05

^a^ one patient did not complete the food frequency questionnaire and was excluded from analysis

^b^ p-value obtained by Kruskal-Wallis test after pairwise comparison of patients with chronic pancreatitis to healthy controls and correction for multiple testing

^c^ p-value obtained by Kruskal-Wallis test after pairwise comparison of patients with liver cirrhosis to healthy controls and correction for multiple testing
^d^ p-value obtained by Kruskal-Wallis test after pairwise comparison of patients with chronic pancreatitis to liver cirrhosis and correction for multiple testing

^e^ Calculation is based on ideal body weight in subjects with ascites, edema, or obesity
