# Supplementary material for: Nutritional status in patients with chronic pancreatitis and liver cirrhosis is related to disease conditions and not dietary habits
Source: Sci Rep. 2024 Feb 26;14:4700. doi: 10.1038/s41598-024-54998-7 (PMC10897307; doi:10.1038/s41598-024-54998-7)
Supplement: Supplementary file 7 — Supplementary Table S7. [file 41598_2024_54998_MOESM7_ESM.docx]

**Supplementary Table 7:** Comparison of micronutrient intake in patients with chronic pancreatitis or liver cirrhosis and healthy controls stratified by sex

|  | **Female** | | | | | |  | **Male** | | | | | | |
| --- | --- | --- | --- | --- | --- | --- | --- | --- | --- | --- | --- | --- | --- | --- |
|  | **Chronic pancreatitis**  **(n=15)** | **Liver cirrhosis**  **(n=26)** | **Healthy controls**  **(n=49)** | **p-value^b^** | **p-value^c^** | **p-value^d^** |  | **Chronic pancreatitis (n=50)^a^** | | **Liver cirrhosis**  **(n=52)** | **Healthy controls**  **(n=45)** | **p-value^b^** | **p-value^c^** | **p-value**^d^ |
| Sodium, mg/d | 1584 (1234) | 1523 (1243) | 1992 (1253) | 0.478 | 0.166 | 1.000 |  | | 2390 (1052) | 1957 (1308) | 2289 (1058) | 1.000 | 0.461 | 0.071 |
| Potassium, mg/d | 2112 (1608) | 2626 (1641) | 2992 (1524) | 0.077 | 0.207 | 1.000 |  | | 3038 (1507) | 2932 (1772) | 3525 (2385) | 0.620 | 0.149 | 1.000 |
| Calcium, mg/d | 710 (726) | 898 (521) | 1030 (781) | 0.260 | 0.822 | 1.000 |  | | 942 (758) | 857 (572) | 1033 (684) | 1.000 | 0.194 | 0.896 |
| Phosphorus, mg/d | 970 (741) | 916 (868) | 1205 | 0.196 | 0.152 | 1.000 |  | | 1397 (623) | 1346 (755) | 1507 (925) | 1.000 | 0.225 | 1.000 |
| Magnesium, mg/d | 319 (135) | 330 (155) | 373 (323) | 0.299 | 0.062 | 1.000 |  | | 372 (156) | 366 (176) | 420 (297) | 0.610 | **0.017** | 0.377 |
| Iron, mg/d | 11 (4) | 12 (5) | 14 (9) | 0.117 | 0.054 | 1.000 |  | | 16 (5) | 13 (7) | 16 (9) | 0.933 | **0.009** | 0.142 |
| Zinc, mg/d | 9 (6) | 10 (8) | 12 (8) | **0.044** | 0.059 | 1.000 |  | | 12 (4) | 12 (9) | 14 (7) | 0.488 | 0.135 | 1.000 |
| Vitamin A, µg/d | 448 (307) | 878 (1387) | 881 (1497) | 0.117 | 1.000 | 0.064 |  | | 3082 (4046) | 1974 (2801) | 1248 (2260) | **0.012** | 0.329 | 0.534 |
| Vitamin E, mg/d | 8 (4) | 8 (6) | 9 (7) | 0.686 | 0.157 | 1.000 |  | | 9 (6) | 8 (6) | 10 (7) | 0.157 | 0.063 | 1.000 |
| Vitamin B1, mg/d | 1 (0) | 1 (0) | 1 (1) | 0.584 | **0.018** | 1.000 |  | | 2 (1) | 2 (1) | 2 (1) | 1.000 | 1.000 | 1.000 |
| Vitamin B2, mg/d | 9 (33) | 15 (36) | 10 (34) | 1.000 | 1.000 | 1.000 |  | | 16 (42) | 10 (26) | 20 (52) | 1.000 | 0.215 | 0.636 |
| Vitamin B6, mg/d | 1 (1) | 1 (1) | 2 (1) | 0.124 | 0.097 | 1.000 |  | | 2 (1) | 2 (1) | 2 (1) | 0.149 | 0.158 | 1.000 |
| Folic acid, µg/d | 167 (122) | 202 (130) | 236 (127) | **0.030** | 0.069 | 1.000 |  | | 222 (189) | 270 (149) | 280 (159) | 0.240 | 0.591 | 1.000 |
| Vitamin B12, µg/d | 3 (2) | 5 (4) | 5 (4) | 0.052 | 1.000 | 0.393 |  | | 9 (7) | 7 (6) | 7 (5) | 0.192 | 1.000 | 0.492 |
| Vitamin C, mg/d | 142 (213) | 153 (222) | 260 (217) | 0.176 | 0.188 | 1.000 |  | | 121 (138) | 150 (148) | 233 (227) | **0.001** | **0.046** | 0.256 |

All data is presented as median (IQR); bold typed numbers indicate p-value < 0.05

^a^ one patient did not complete the food frequency questionnaire and was excluded from analysis

^b^ p-value obtained by Kruskal-Wallis test after pairwise comparison of patients with chronic pancreatitis to healthy controls and correction for multiple testing

^c^ p-value obtained by Kruskal-Wallis test after pairwise comparison of patients with liver cirrhosis to healthy controls and correction for multiple testing

^d^ p-value obtained by Kruskal-Wallis test after pairwise comparison of patients with chronic pancreatitis to liver cirrhosis and correction for multiple testing
