# Supplementary material for: Nutritional status in patients with chronic pancreatitis and liver cirrhosis is related to disease conditions and not dietary habits
Source: Sci Rep. 2024 Feb 26;14:4700. doi: 10.1038/s41598-024-54998-7 (PMC10897307; doi:10.1038/s41598-024-54998-7)
Supplement: Supplementary file 8 — Supplementary Table S8. [file 41598_2024_54998_MOESM8_ESM.docx]

**Supplementary Table 8:** Comparison of food group consumption between patients with chronic pancreatitis and liver cirrhosis and healthy controls

|  | **Chronic pancreatitis**  **(n=65)^a^** | **Liver cirrhosis**  **(n=78)** | **Healthy controls**  **(n=94)** | **p-value^b^** | **p-value^c^** | **p-value^d^** |
| --- | --- | --- | --- | --- | --- | --- |
| Water, ml/d | 1050 (1334) | 900 (600) | 1200 (3000) | 0.277 | **0.017** | 1.000 |
| Light drinks, ml/d | 0 (0) | 0 (0) | 0 (0) | 1.000 | 0.192 | 0.792 |
| Lemonade, ml/d | 38 (202) | 157 (380) | 58 (199) | 0.515 | 0.271 | **0.013** |
| Coffee, ml/d | 300 (589) | 38 (295) | 300 (450) | **0.044** | **<0.001** | **0.001** |
| Tea, ml/d | 150 (345) | 150 (268) | 59 (288) | 0.168 | 0.398 | 1.000 |
| Alcoholic beverages, ml/d | 0 (29) | 0 (76) | 86 (167) | **<0.001** | **<0.001** | 1.000 |
| Beer, ml/d | 0 (15) | 0 (0) | 29 (124) | **0.001** | **<0.001** | 1.000 |
| Non-alcoholic beer, ml/d | 0 (13) | 0 (0) | 0 (18) | 0.853 | **<0.001** | **0.017** |
| Wine & sparkling wine, ml/d | 0 (0) | 0 (0) | 0 (50) | **<0.001** | **<0.001** | 1.000 |
| High-percentage alcoholic drinks, ml/d | 0 (0) | 0 (0) | 0 (1) | **0.003** | 1.000 | 1.000 |
| Cocktails, ml/d | 0 (0) | 0 (0) | 0 (7) | **0.002** | **<0.001** | 0.753 |
| White bread, g/d | 100 (131) | 50 (84) | 26 (55) | **<0.001** | 0.067 | **0.005** |
| Whole grain products, g/d | 12 (50) | 11 (50) | 50 (89) | **<0.001** | **<0.001** | 1.000 |
| Cereals & cornflakes, g/d | 0 (5) | 0 (0) | 1 (13) | **0.033** | **<0.001** | 0.916 |
| Fruits & vegetables, g/d | 241 (296) | 271 (359) | 428 (412) | **<0.001** | **<0.001** | 1.000 |
| Rice & noodles, g/d | 17 (23) | 15 (39) | 27 (37) | 0.389 | 0.074 | 1.000 |
| Boiled potatoes, g/d | 88 (100) | 75 (94) | 38 (72) | **<0.001** | 0.409 | 0.050 |
| Roast potatoes, g/d | 3 (13) | 0 (7) | 3 (13) | 1.000 | 0.091 | 0.140 |
| Low-fat dairy products, g/d | 0 (0) | 0 (0) | 0 (0) | 1.000 | 1.000 | 1.000 |
| Dairy products, g/d | 105 (156) | 133 (202) | 103 (123) | 1.000 | 0.096 | 0.144 |
| Eggs, g/d | 13 (14) | 13 (19) | 26 (17) | 0.361 | 0.274 | 1.000 |
| Low-fat sausages, g/d | 4 (9) | 0 (4) | 4 (4) | 0.053 | 0.053 | **0.011** |
| High-fat sausages, g/d | 20 (37) | 10 (36) | 9 (19) | **0.018** | **0.033** | 1.000 |
| Meat & poultry, g/d | 46 (57) | 38 (55) | 46 (59) | 0.425 | 1.000 | 0.115 |
| Fish, g/d | 13 (16) | 8 (23) | 16 (21) | 1.000 | **0.019** | 0.314 |
| Butter & margarine, g/d | 10 (15) | 10 (15) | 5 (7) | **<0.001** | **0.006** | 0.301 |
| Fast Food, g/d | 16 (26) | 5 (26) | 30 (40) | 0.148 | **<0.001** | 0.078 |
| Crisps, salty pastries, crackers, g/d | 0 (2) | 0 (4) | 1 (4) | 0.271 | 0.274 | 1.000 |
| Desserts & sweet spreads, g/d | 91 (133) | 55 (95) | 73 (65) | 1.000 | 0.667 | 0.364 |
| Nuts, g/d | 0 (5) | 0 (1) | 2 (6) | **0.012** | **<0.001** | **0.012** |

All data is presented as median (IQR); bold typed numbers indicate p-value < 0.05

^a^ one patient did not complete the food frequency questionnaire and was excluded from analysis

^b^ p-value obtained by Kruskal-Wallis test after pairwise comparison of patients with chronic pancreatitis to healthy controls after correction for multiple testing

^c^ p-value obtained by Kruskal-Wallis test after pairwise comparison of patients with liver cirrhosis to healthy controls after correction for multiple testing

^d^ p-value obtained by Kruskal-Wallis test after pairwise comparison of patients with chronic pancreatitis to patients with liver cirrhosis after correction for multiple testing
