# Supplementary material for: Nutritional status in patients with chronic pancreatitis and liver cirrhosis is related to disease conditions and not dietary habits
Source: Sci Rep. 2024 Feb 26;14:4700. doi: 10.1038/s41598-024-54998-7 (PMC10897307; doi:10.1038/s41598-024-54998-7)
Supplement: Supplementary file 9 — Supplementary Table S9. [file 41598_2024_54998_MOESM9_ESM.docx]

**Supplementary Table 9:** Comparison of food group consumption between female patients with chronic pancreatitis and liver cirrhosis and healthy controls

|  | **Chronic pancreatitis**  **(n=15)** | **Liver cirrhosis**  **(n=26)** | **Healthy controls**  **(n=49)** | **p-value^a^** | **p-value^b^** | **p-value^c^** |
| --- | --- | --- | --- | --- | --- | --- |
| Water, ml/d | 900 (1763) | 900 (600) | 1200 (3900) | 0.171 | **0.014** | 1.000 |
| Light drinks, ml/d | 0 (0) | 0 (0) | 0 (7) | 0.640 | 0.918 | 1.000 |
| Lemonade, ml/d | 15 (43) | 100 (198) | 36 (147) | 0.180 | 0.451 | **0.016** |
| Coffee, ml/d | 225 (561) | 150 (287) | 450 (300) | 0.168 | **<0.001** | 0.663 |
| Tea, ml/d | 316 (523) | 150 (247) | 96 (283) | 0.205 | 1.000 | 0.680 |
| Alcoholic beverages, ml/d | 0 (5) | 0 (8) | 54 (107) | **<0.001** | **<0.001** | 1.000 |
| Beer, ml/d | 0 (0) | 0 (0) | 0 (29) | **0.039** | **0.001** | 1.000 |
| Non-alcoholic beer, ml/d | 0 (9) | 0 (0) | 0 (12) | 0.705 | **0.004** | 0.475 |
| Wine & sparkling wine, ml/d | 0 (0) | 0 (0) | 0 (45) | **<0.001** | **<0.001** | 1.000 |
| High-percentage alcoholic drinks, ml/d | 0 (0) | 0 (0) | 0 (1) | 0.090 | 0.269 | 1.000 |
| Cocktails, ml/d | 0 (0) | 0 (0) | 0 (7) | **0.005** | **0.001** | 1.000 |
| White bread, g/d | 50 (90) | 25 (89) | 15 (48) | **0.013** | 0.221 | 0.695 |
| Whole grain products, g/d | 21 (47) | 11 (75) | 50 (89) | 0.054 | 0.143 | 1.000 |
| Cereals & cornflakes, g/d | 4 (13) | 0 (0) | 1 (13) | 1.000 | **0.004** | **0.016** |
| Fruits & vegetables, g/d | 339 (398) | 220 (231) | 434 (490) | 0.234 | **0.002** | 0.961 |
| Rice & noodles, g/d | 27 (49) | 11 (31) | 17 (31) | 1.000 | 0.652 | 0.396 |
| Boiled potatoes, g/d | 88 (100) | 38 (96) | 38 (72) | 0.141 | 1.000 | 0.863 |
| Roast potatoes, g/d | 3 (7) | 0 (5) | 1 (5) | 0.796 | 1.000 | 0.927 |
| Low-fat dairy products, g/d | 0 (0) | 0 (0) | 0 (0) | 1.000 | 1.000 | 1.000 |
| Dairy products, g/d | 92 (127) | 151 (262) | 116 (156) | 1.000 | 0.280 | 0.383 |
| Eggs, g/d | 13 (16) | 13 (22) | 13 (17) | 0.256 | 0.337 | 1.000 |
| Low-fat sausages, g/d | 5 (2) | 0 (9) | 2 (4) | 0.578 | 1.000 | 1.000 |
| High-fat sausages, g/d | 1 (4) | 10 (17) | 4 (20) | 0.371 | 0.103 | **0.008** |
| Meat & poultry, g/d | 45 (49) | 21 (40) | 38 (47) | 0.866 | 0.690 | 1.000 |
| Fish, g/d | 18 (20) | 7 (21) | 16 (20) | 1.000 | 0.134 | 0.381 |
| Butter & margarine, g/d | 9 (7) | 8 (18) | 5 (7) | 0.077 | 0.063 | 1.000 |
| Fast Food, g/d | 11 (18) | 5 (25) | 15 (31) | 0.346 | **0.017** | 1.000 |
| Crisps, salty pastries, crackers, g/d | 1 (2) | 0 (4) | 1 (4) | 1.000 | 0.790 | 1.000 |
| Desserts & sweet spreads, g/d | 79 (101) | 51 (128) | 66 (54) | 1.000 | 1.000 | 1.000 |
| Nuts, g/d | 1 (3) | 0 (0) | 2 (6) | 0.182 | **<0.001** | 0.298 |

All data is presented as median (IQR); bold typed numbers indicate p-value < 0.05
^a^ p-value obtained by Kruskal-Wallis test after pairwise comparison of patients with chronic pancreatitis to healthy controls after correction for multiple testing

^b^ p-value obtained by Kruskal-Wallis test after pairwise comparison of patients with liver cirrhosis to healthy controls after correction for multiple testing

^c^ p-value obtained by Kruskal-Wallis test after pairwise comparison of patients with chronic pancreatitis to patients with liver cirrhosis after correction for multiple testing
