# Supplementary material for: Nutritional status in patients with chronic pancreatitis and liver cirrhosis is related to disease conditions and not dietary habits
Source: Sci Rep. 2024 Feb 26;14:4700. doi: 10.1038/s41598-024-54998-7 (PMC10897307; doi:10.1038/s41598-024-54998-7)
Supplement: Supplementary file 10 — Supplementary Table S10. [file 41598_2024_54998_MOESM10_ESM.docx]

**Supplementary Table 10:** Comparison of food group consumption between male patients with chronic pancreatitis and liver cirrhosis and healthy controls

|  | **Chronic pancreatitis**  **(n=50)^a^** | **Liver cirrhosis**  **(n=52)** | **Healthy controls**  **(n=45)** | **p-value^b^** | **p-value^c^** | **p-value^d^** |
| --- | --- | --- | --- | --- | --- | --- |
| Water, ml/d | 1200 (1347) | 900 (600) | 900 (1400) | 1.000 | 1.000 | 1.000 |
| Light drinks, ml/d | 0 (0) | 0 (0) | 0 (0) | 1.000 | 0.932 | 0.239 |
| Lemonade, ml/d | 49 (248) | 186 (455) | 100 (368) | 0.644 | 0.178 | 1.000 |
| Coffee, ml/d | 300 (587) | 32 (150) | 300 (466) | 0.731 | **<0.001** | **0.002** |
| Tea, ml/d | 150 (296) | 150 (268) | 32 (188) | 0.179 | 0.162 | 1.000 |
| Alcoholic beverages, ml/d | 0 (82) | 0 (380) | 130 (257) | **<0.001** | **<0.001** | 1.000 |
| Beer, ml/d | 0 (0) | 0 (59) | 88 (143) | **<0.001** | **<0.001** | 1.000 |
| Non-alcoholic beer, ml/d | 0 (25) | 0 (0) | 0 (29) | 1.000 | **0.019** | 0.071 |
| Wine & sparkling wine, ml/d | 0 (0) | 0 (0) | 9 (22) | **<0.001** | **<0.001** | 1.000 |
| High-percentage alcoholic drinks, ml/d | 0 (0) | 0 (0) | 1 (2) | **0.006** | 0.061 | 1.000 |
| Cocktails, ml/d | 0 (0) | 0 (0) | 0 (4) | 0.323 | **0.014** | 0.640 |
| White bread, g/d | 111 (147) | 61 (104) | 50 (79) | **0.002** | 1.000 | **0.015** |
| Whole grain products, g/d | 10 (50) | 11 (50) | 50 (98) | **0.008** | **0.005** | 1.000 |
| Cereals & cornflakes, g/d | 0 (0) | 0 (2) | 1 (25) | **0.017** | 0.051 | 1.000 |
| Fruits & vegetables, g/d | 220 (240) | 316 (367) | 407 (393) | **0.002** | 0.127 | 0.422 |
| Rice & noodles, g/d | 17 (16) | 24 (40) | 32 (41) | **0.014** | **0.044** | 1.000 |
| Boiled potatoes, g/d | 88 (109) | 82 (102) | 38 (50) | **0.024** | 1.000 | 0.128 |
| Roast potatoes, g/d | 4 (13) | 0 (8) | 13 (10) | 0.207 | **0.003** | 0.428 |
| Low-fat dairy products, g/d | 0 (0) | 0 (0) | 0 (0) | 1.000 | 1.000 | 1.000 |
| Dairy products, g/d | 107 (174) | 124 (200) | 83 (108) | 1.000 | 0.190 | 0.610 |
| Eggs, g/d | 13 (13) | 13 (19) | 26 (17) | 0.574 | 0.549 | 1.000 |
| Low-fat sausages, g/d | 4 (12) | 0 (4) | 4 (8) | **0.024** | **0.002** | 1.000 |
| High-fat sausages, g/d | 25 (51) | 10 (36) | 9 (28) | **0.011** | 0.676 | 0.231 |
| Meat & poultry, g/d | 55 (58) | 46 (61) | 48 (61) | 1.000 | 1.000 | 0.397 |
| Fish, g/d | 11 (13) | 9 (26) | 19 (23) | 0.779 | 0.124 | 1.000 |
| Butter & margarine, g/d | 15 (15) | 10 (15) | 8 (10) | **0.006** | 0.240 | 0.485 |
| Fast Food, g/d | 17 (36) | 6 (31) | 39 (62) | 0.108 | **<0.001** | 0.160 |
| Crisps, salty pastries, crackers, g/d | 0 (2) | 0 (4) | 1 (4) | 0.334 | 0.582 | 1.000 |
| Desserts & sweet spreads, g/d | 97 (142) | 58 (90) | 74 (91) | 1.000 | 0.578 | 0.363 |
| Nuts, g/d | 0 (5) | 0 (1) | 2 (6) | 0.110 | **<0.001** | 0.082 |

All data is presented as median (IQR); bold typed numbers indicate p-value < 0.05

^a^ one patient did not complete the food frequency questionnaire and was excluded from analysis

^b^ p-value obtained by Kruskal-Wallis test after pairwise comparison of patients with chronic pancreatitis to healthy controls after correction for multiple testing

^c^ p-value obtained by Kruskal-Wallis test after pairwise comparison of patients with liver cirrhosis to healthy controls after correction for multiple testing

^d^ p-value obtained by Kruskal-Wallis test after pairwise comparison of patients with chronic pancreatitis to patients with liver cirrhosis after correction for multiple testing
